# *In silico* analysis of the core signaling proteome from the barley powdery mildew pathogen (*Blumeria graminis* f.sp. *hordei*)

**DOI:** 10.1186/1471-2164-15-843

**Published:** 2014-10-02

**Authors:** Stefan Kusch, Nahal Ahmadinejad, Ralph Panstruga, Hannah Kuhn

**Affiliations:** Unit of Plant Molecular Cell Biology, Institute for Biology I, RWTH Aachen University, Worringerweg 1, 52056 Aachen, Germany; Institute of Biochemistry and Molecular Biology, University of Bonn, Nussallee 11, 53115 Bonn, Germany

**Keywords:** *Blumeria graminis*, cAMP, Calcium, GPCR, Heterotrimeric G protein, MAP kinase, Powdery mildew, Small GTPase, Transcription factors

## Abstract

**Background:**

Compared to other ascomycetes, the barley powdery mildew pathogen *Blumeria graminis* f.sp. *hordei* (*Bgh*) has a large genome (ca. 120 Mbp) that harbors a relatively small number of protein-coding genes (ca. 6500). This genomic assemblage is thought to be the result of numerous gene losses, which likely represent an evolutionary adaptation to a parasitic lifestyle in close association with its host plant, barley (*Hordeum vulgare*). Approximately 8% of the *Bgh* genes are predicted to encode virulence effectors that are secreted into host tissue and/or cells to promote pathogenesis; the remaining proteome is largely uncharacterized at present.

**Results:**

We provide a comparative analysis of the conceptual *Bgh* proteome, with an emphasis on proteins with known roles in fungal development and pathogenicity, for example heterotrimeric G proteins and G protein coupled receptors; components of calcium and cAMP signaling; small monomeric GTPases; mitogen-activated protein cascades and transcription factors. The predicted *Bgh* proteome lacks a number of proteins that are otherwise conserved in filamentous fungi, including two proteins that are required for the formation of anastomoses (somatic hyphal connections). By contrast, apart from minor modifications, all major canonical signaling pathways are retained in *Bgh*. A family of kinases that preferentially occur in pathogenic species of the fungal clade Leotiomyceta is unusually expanded in *Bgh* and its close relative, *Blumeria graminis* f.sp. *tritici*.

**Conclusions:**

Our analysis reveals characteristic features of the proteome of a fungal phytopathogen that occupies an extreme habitat: the living plant cell.

**Electronic supplementary material:**

The online version of this article (doi:10.1186/1471-2164-15-843) contains supplementary material, which is available to authorized users.

## Background

Powdery mildew is a prevalent disease of many higher plant species that is caused by ascomycetes of the order Erysiphales [1]. Members of the order Erysiphales have an obligate biotrophic lifestyle, i.e. they can only grow and propagate on living plant tissue; *in vitro* propagation and genetic manipulation (transformation) are currently impossible. While some powdery mildew fungi have a wide host range and can infect a broad spectrum of plant species, others have a narrow host range and can often infect only a single plant species. A well-known representative of the latter is *Blumeria graminis* f.sp. *hordei* (*Bgh*), the causal agent of the barley powdery mildew disease, which exclusively colonizes barley (*Hordeum vulgare*). Grass powdery mildews of the genus *Blumeria* are serious phytopathogens that cause considerable yield losses in agricultural settings [2].

We previously sequenced and partially assembled the genomes of the three powdery mildew species *Bgh* (isolate DH14), *Erysiphe pisi* (the pea powdery mildew pathogen) and *Golovinomyces orontii* (one of several powdery mildew species that are able to colonize the model plant species *Arabidopsis thaliana*; [3]). Recently, the genome sequence of a fourth powdery mildew species (*Blumeria graminis* f.sp. *tritici* (*Bgt*), the wheat powdery mildew pathogen), has been published [4] and additional *Bgh* isolates have been sequenced [5]. These studies show that in comparison to other ascomycetes, powdery mildew genomes are unusually large (ca. 120–160 Mbp). This is chiefly due to a genome structure typified by the presence of numerous nested retrotransposon copies with few interspersed genes. Haplotype structure of the *Bgh* and *Bgt* genomes reveals a mosaic pattern of alternating homomorphic and polymorphic blocks, which has been interpreted as an indication of frequent asexual and rare sexual reproduction [4, 5].

Despite their huge size, powdery mildew genomes are characterized by a comparatively small number of conventional protein-coding genes. For example, the genomes of *Bgh* and *Bgt* each harbor ~6,500 annotated genes [3, 4]. This is at the lower end for ascomycete phytopathogens [6]. The reduction in gene content is partly due to a drastic decrease in genes coding for cell wall-degrading enzymes (carbohydrate-active enzymes; CAZys) and secondary metabolite biosynthesis enzymes, which is consistent with their parasitic obligate biotrophic lifestyle. In addition, powdery mildews have lost multiple genes that are otherwise conserved in ascomycetes, ranging from unicellular yeasts to filamentous fungi. These genes comprise, amongst others, enzymes required for nitrate and sulfate assimilation and biosynthesis of thiamine (vitamin B1) [3]. Interestingly, similar gene losses can also be seen in the genomes of the very distantly related rust fungi (basidiomycetes) and downy mildews (oomycetes), which also possess obligate biotrophic lifestyles, suggesting that the reduction in gene content represents lifestyle-associated convergent evolution [3].

It can be assumed that the list of common fungal genes found to be missing in *Bgh* is incomplete. First, very strict criteria were applied to identify missing genes in *Bgh*. Proteins belonging to different families, but sharing common domains, may thus have escaped detection. Second, the starting point for the analysis was the well-annotated proteome of yeast, which is a unicellular fungus. Therefore, all proteins that are specific to filamentous fungi were not considered. Third, missing family members cannot be identified by this approach. Fourth, species-specific gene losses in individual powdery mildew species might well have been overlooked, since absence of the genes in all three powdery mildew species under consideration (*Bgh*, *G. orontii* and *E. pisi*) was a criterion of the bioinformatic pipeline. We therefore expect that careful manual analysis would uncover additional genes missing in the *Bgh* genome.

As other phytopathogens, powdery mildew fungi are thought to deploy a suite of secreted effector proteins for host cell manipulation. Genome-wide analysis revealed 491 Candidate Secreted Effector Proteins (CSEPs) that are encoded by the *Bgh* genome [7]. CSEPs represent comparatively small polypeptides that harbor a predicted N-terminal signal peptide for secretion, lack sequence-relatedness to known proteins in the NCBI database and often show evidence of diversifying selection [7]. Notably, a considerable number of the *Bgh* CSEPs show predicted structural similarities to microbial ribonucleases [7]. A similar, though slightly higher, number (602) of effector-encoding genes was found in *Bgt* [4]. Interestingly, 437 CSEPs are shared between *Bgh* and *Bgt,* and appear to represent *Blumeria*-specific genes, since there is no evidence for their existence outside this genus. First functional studies have begun to elucidate the contribution of individual CSEPs to plant colonization [8, 9]. Besides the CSEPs, the *Bgh* genome encodes a huge suite of unconventional effector candidates that lack an amino-terminal secretion signal and are physically associated and seemingly coevolved with LINE-1 retrotransposons [10]. These proteins were first recognized upon cloning of the avirulence genes matching the barley *Mlk1* and *Mla10* resistance genes [11] and are therefore also designated putative effectors with similarity to AVRK1 and AVRA10 (EKAs).

Despite extensive characterization of genomic organization [3], haplotype structure [5] and effector content [7, 10] of the *Bgh* genome, little attention has been paid to its core proteome. Here we provide a global characterization of the full *Bgh* proteome and analyze in detail selected protein families with well-known roles in fungal development and pathogenicity.

## Results and discussion

### Global characterization of the conceptual *Bgh*proteome

The annotated *Bgh* isolate DH14 genome (v3.0) comprises 6,470 genes, including ca. 250 partial genes (5′- or 3′- truncated) and/or genes that are split on two contigs (http://www.blugen.org/). In the course of this analysis we annotated an additional 25 genes, of which one (mating pheromone receptor Ste3) was not represented by a genomic contig of isolate DH14 but deduced from RNA sequencing data from *Bgh* isolate K1 [5]. Altogether, this resulted in a total of 6,495 genes, which form the basis for the present study (Additional file 1: Table S1, sheet “Summary”). We used the respective predicted amino acid sequences to perform a number of bioinformatic analyses to characterize the proteome of *Bgh* DH14.

**Figure 1:**
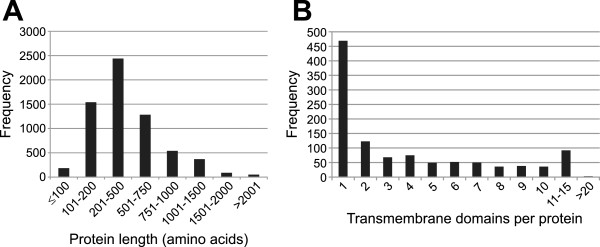
**Global parameters of the** ***Bgh*** **proteome.** Histograms showing the frequency distribution of protein length **(A)** and the frequency distribution of transmembrane domains per protein **(B)**.

The size of the predicted *Bgh* proteins is 483 ± 372 (mean ± standard deviation) amino acids, which is close to the average protein size of 487 amino acids recently reported for fungi [12]. A major proportion (37.5%) of the *Bgh* proteins lie between 250 and 500 amino acids in size (Figure 1A). Few proteins are larger than 1,500 amino acids (509, ca. 8%) or smaller than 100 amino acids (183, ca. 3%). According to the current annotation, the smallest protein, with 25 amino acids, is the 60S ribosomal protein L41 (EBI/GenBank accession number CEA17194). The largest protein (accession number CCU82148), composed of 4,817 amino acids, is a homolog of midasin, a huge AAA ATPase distantly related to the motor protein dynein.

TMHMM2.0 analysis indicated that 1,091 *Bgh* proteins harbor at least one transmembrane (TM) domain (Additional file 1: Table S1, sheet “Summary”). The range of predicted TM domains per protein is between one (469 proteins) and 24 (one protein), with few proteins (95) having more than ten predicted TM domains (Figure 1B). Similar to this calculation, the extrapolation of experimentally determined subcellular localization data suggests the presence of somewhat more than 1,000 integral membrane proteins in the unicellular ascomycete *Saccharomyces cerevisiae* [13]. Analysis with SignalP4.1 revealed that 731 proteins (~11% of the predicted *Bgh* proteome) harbor a predicted signal peptide for secretion at their amino terminus (Additional file 1: Table S1, sheet “Summary”). In absolute numbers this value is at the lower end for SignalP-predicted proteins of the fungal subphylum Pezizomycotina; however, in relation to the size of the proteome it is rather at the upper end [14]. These putatively secreted *Bgh* proteins comprise 421 of the 491 previously identified CSEPs. The seeming discrepancy for the remaining 70 CSEPs results from prediction algorithm changes, in the now used SignalP4.1 version, compared to the formerly used version 3.0. These instances may thus comprise borderline cases for which signal peptide prediction is ambiguous. However, analysis with the SignalP4.1 algorithm corroborated the presence of an N-terminal signal peptide for 86% of the previously identified CSEPs and allowed the identification of 42 additional secreted effector candidates that were differentially classified or overlooked before (Additional file 1: Table S1, sheet “Additional effector candidates”). In conclusion, this analysis shows that CSEPs account for more than half of the *Bgh* proteins with a predicted signal peptide.

BLASTP searches, in the context of BLAST2GO analysis, revealed 5,665 *Bgh* proteins with one or more hits in the NCBI database (E value <1e-06), while 830 proteins had no significant hits (E value ≥ 1e-06; Additional file 1: Table S1, sheet “No BLAST hit”) at the time of analysis. *Botryotinia fuckeliana*, *Glarea lozoyensis*, *Marssonina brunnea*, *Fusarium oxysporum* and *Sclerotinia sclerotiorum* were the species that yielded the greatest total number of BLAST hits (Additional file 2: Figure S1a); whereas *Marssonina brunnea*, *Glarea lozoyensis* and *Botryotinia fuckeliana* were the species that yielded most top BLAST hits for the *Bgh* query sequences (Additional file 2: Figure S1b).

We used Markov clustering (MCL) to group the 6,495 predicted *Bgh* proteins into polypeptide families (see Methods for details). This type of analysis revealed that the *Bgh* genome encodes 619 protein families, ranging in size from two to 230 members, plus 3,758 singletons. Most protein families are small (544 families with 2–5 members) or medium-sized (68 families with 6–25 members), and only seven families comprise more than 25 members (Additional file 3: Table S2). EKA-like avirulence proteins, six different CSEP families, kinases, WD40 domain proteins, ATP-dependent RNA helicases, mitochondrial carrier proteins and AAA ATPases comprise the protein families with the highest number of members in the *Bgh* proteome (Table 1). This outcome is largely consistent with the analysis of protein domains by InterProScan, which revealed that WD40 and kinase domains are amongst the most prevalent domains in *Bgh* proteins (Additional file 4: Table S3). WD40 domains exhibit a β-propeller architecture and are amongst the most abundant domains in eukaryotic organisms. They are typically involved in mediating protein-protein or protein-DNA interactions [15]. Notably, seven of the 20 largest protein families appear to be, largely, *Blumeria*-specific. These include the EKA avirulence proteins and the six largest CSEP families (Table 1).

**Table 1:** **The 20 largest** ***Bgh*** **protein families according to MCL analysis**

Number of family members	Protein function
230	EKA family (effectors paralogous to AVRK1 and AVRA10)
150	CSEP (family 1)
75	Serine/threonine kinase
70	Fungus-specific tyrosine kinase
57	CSEP (family 2; RNAse domain)
41	WD40 domain protein
27	Mitochondrial transporter/carrier protein
25	CSEP (family 3; RNAse domain)
25	ATP-dependent RNA helicase
24	Reverse transcriptase/endonuclease
20	CSEP (family 4)
20	Bromodomain-containing ATP-dependent chromatin remodeling factor (DNA repair protein/helicase)
20	30 kDa heat shock protein
19	AAA family ATPase
17	Small monomeric GTPase
16	RNA recognition motif-containing protein
15	(Short-chain) dehydrogenase (oxidoreductase)
15	Ubiquitin conjugating enzyme (E2)
14	CSEP (family 5)
13	CSEP (family 6)

### Predicted subcellular localization of *Bgh*proteins

We performed an analysis of potential subcellular localization for the 6,495 conceptual *Bgh* proteins using ProtComp (Version 9.0), which combines several methods for prediction of protein localization (see Methods for details). In the context of our study we considered the outcome of “neural network analysis” and the “integral final score”, the latter condensing the results of four different prediction methods into a final mark. Both approaches yielded similar prediction profiles (Additional file 5: Figure S2), with consistent predictions of subcellular localization for 4,724 proteins and conflicting predictions for 1,771 proteins. Results of the ProtComp analysis further support a secretory pathway route for a subset of the known CSEPs and additional effector candidates, since 200 out of the 491 previously published CSEPs and 17 out of the 42 additional CSEPs proposed in the context of this work, were classified as “extracellular (secreted)” by both the neural network method and the integral final score (Additional file 1: Table S1, sheets “CSEPs” and “Additional effector candidates”).

### A family of fungus-specific kinases is expanded in *Bgh*and *Bgt*

**Figure 2:**
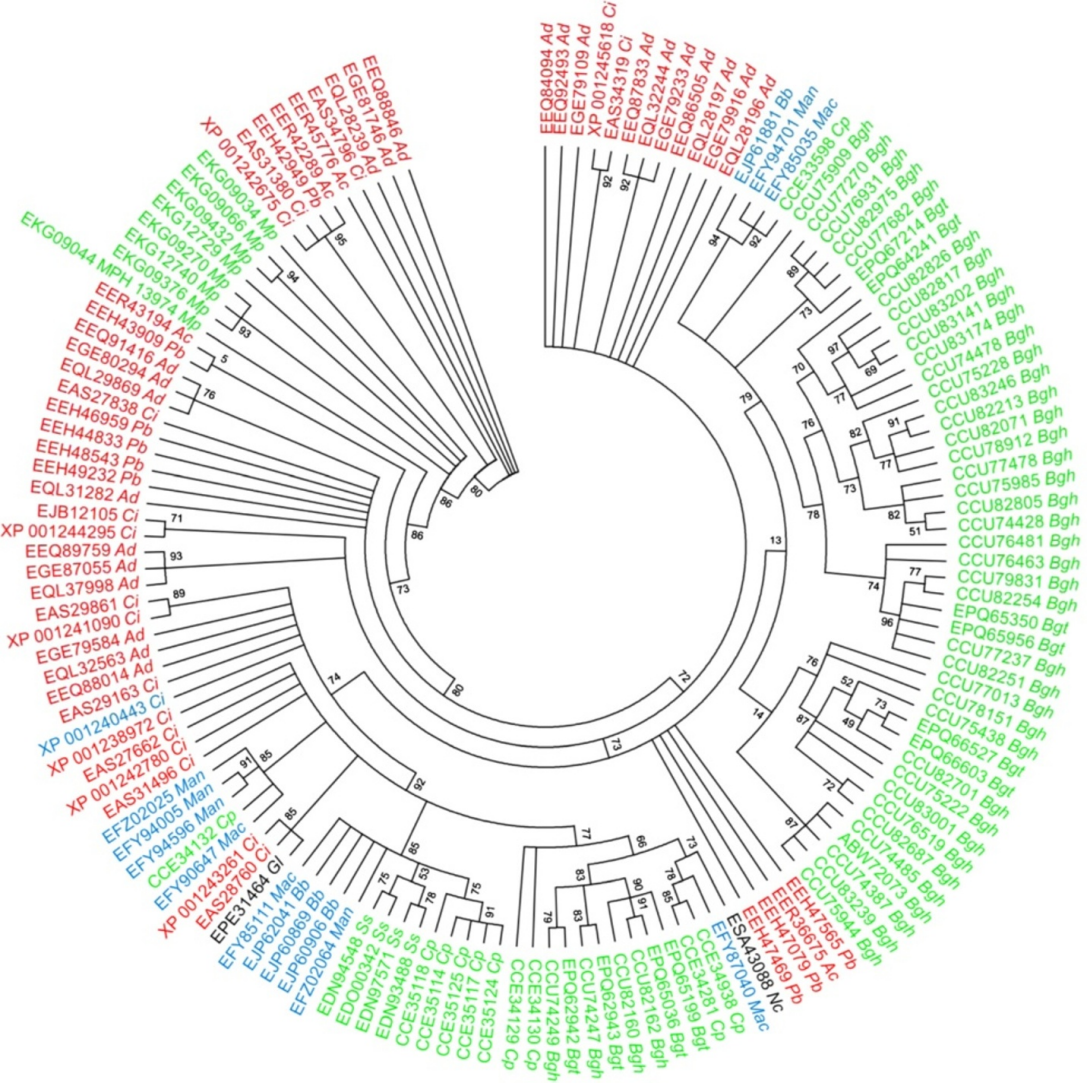
**Cladogram of the fungus-specific kinase family.** The tree integrates BLASTP results (E value <1e-20) using the *Bgh* protein CCU82254 as a query sequence. Phylogenetic analysis was performed with the tool Phylogeny.fr (http://phylogeny.lirmm.fr/phylo_cgi/index.cgi) with the following workflow: Protein sequences were aligned using MUSCLE3.7 with default settings. Poorly aligned positions and divergent regions were eliminated by Gblocks 0.91b with default settings. Presumably incomplete sequences (lacking an N-terminal methionine or < 250 amino acids) as well as sequences that were not properly aligned after curation were removed from the alignment. The phylogenetic tree was calculated by PhyML3.0 with computation of bootstrap values (100 replicates). MEGA6 was used to render the cladogram. Condensation of branches was performed with a cut off of for bootstrap values <2. Gene bank accessions and taxa are given in green (plant pathogenic fungi), red (human pathogenic fungi), blue (insect pathogenic fungi) and black (saprophytic fungi). *Ac*, *Ajellomyces capsulatus*; *Ad*, *Ajellomyces dermatitidis;* *Bb*, *Beauveria bassiana*; *Bgh*, *Blumeria graminis* f. sp. *hordei*; *Bgt*, *Blumeria graminis* f. sp. *tritici*; *Ci*, *Coccidioides immitis*; *Cp*, *Claviceps purpurea*; *Fp*, *Fusarium pseudograminearum*, *Gl*, *Glarea lozoyensis*; *Mac*, *Metarhizium acridum*; *Man*, *Metarhizium anisopliae*; *Mp*, *Macrophomina phaseolina*, *Nc*, *Neurospora crassa*; *Pb*, *Paracoccidioides brasiliensis*; *Ss*, *Sclerotinia sclerotiorum*.

We noted the drastic expansion of a family of fungus-specific kinases in *Bgh* compared to other fungi (family 4, Table 1). This type of kinases appears to be largely restricted to the fungal clade of Leotiomyceta; outside of this taxonomic division only few kinases with low sequence similarity can be found (no BLAST hit with an E value <1e-20). Within the Leotiomyceta, the kinases appear to be most prevalent in pathogenic species representing different taxonomic classes, in particular phytopathogens, but also some facultative human and insect pathogens. By contrast, saprophytic fungi seem to either lack these kinases (for example *Penicillium chrysogenum* or *S. cerevisiae*) or only harbor single/few copies thereof (for example *Aspergillus niger*, *Glarea lozoyensis* or *Neurospora crassa*; Additional file 6: Table S4). Nevertheless, some phytopathogenic fungal species lack these kinases (for example *Botryotinia fuckeliana*, *Colletotrichum higginsianum*, *Fusarium oxysprum*, *Magnaporthe oryzae* and *Marssonina brunnea*), indicating that their presence is not a strict prerequisite for a pathogenic lifestyle. The presence/absence pattern of these proteins in fungal taxa suggests several independent losses of genes encoding this kinase type within the Leotiomyceta, concomitant with a convergent expansion of this gene family in a subset of pathogenic species. This notion is further supported by phylogenetic analysis of this protein family, which in most cases revealed species-/clade-specific grouping rather than paralog-specific clustering of the respective protein sequences (Figure 2). Such a phylogenetic pattern is indicative of species-specific expansions of the kinase family subsequent to fungal speciation events.

According to our sequence similarity database searches, the *Bgh* genome encompasses the highest number of genes encoding this kinase type (70 members), while the *Bgt* genome encodes 25 members of this protein family (Additional file 6: Table S4). The difference in the size of this kinase family between these two closely related *formae speciales* is striking and may relate to problems with genome annotation (see below). Alternatively, this finding could indicate that the kinase genes evolved differently in *Bgh* and *Bgt*. Notably, the genomes of the powdery mildews infecting dicotyledonous plant species, *E. pisi* and *G. orontii*, each seem to contain only one member of this kinase type (corresponding to *Bgh* CCU83175; E values 7e-13 and 1e-24, respectively; Additional file 1: Table S1, sheet “Fungus-specific kinase”). In *Bgh*, many members of the *Bgh* kinase family reside on comparatively small contigs of the current DH14 genome assembly (41 of the 70 contigs are <10 kb; http://www.blugen.org/). In most cases these contigs lack other genes (only 20 of the 70 contigs harbor one or more additional genes; http://www.blugen.org/). This scenario suggests that these genes are mostly localized/isolated within highly repetitive genomic regions that are difficult to assemble. The full-length sequences of these kinases are typically 500–1000 amino acids in length. Owing to the small size of the corresponding genomic contigs several members are N- and/or C-terminally truncated. Thus, the actual number of these kinases might be lower than indicated in Additional file 6: Table S4, since pairs of N- and C-terminally truncated proteins may represent the same protein.

The predicted kinase domain resides in the carboxy-terminal half of the proteins. Twenty-one of the 70 *Bgh* paralogs are annotated with InterProScan domain IPR008266 (Tyrosin protein kinase, active site; Additional file 1: Table S1, sheet “Fungus-specific kinase”), suggesting that these proteins possess tyrosine kinase activity. However, it is generally thought that fungi lost tyrosine kinases in the course of evolution [16, 17] and that only subgroups of the basidiomycetes encode kinases that are closely related to tyrosine kinases [17]. Consistent with this belief, virtual structure prediction, *via* the protein fold recognition server Phyre^2^ (http://www.sbg.bio.ic.ac.uk/phyre2/html/page.cgi?id=index), revealed highest structural similarity of 27 of these kinases to the human serine/threonine kinase vaccinia-related kinase 1 (vrf1). The contradictory *in silico* predictions prevent, at present, the reliable classification of this kinase family regarding its substrate specificity; however, the lack of additional tyrosine kinase specific sequence motifs [17] rather suggests that these proteins may have no tyrosine kinase activity. Apart from the signatures mentioned above, no other known protein domains can be found in these kinases. Interestingly, the genome of the obligate biotrophic wheat rust pathogen (*Puccinia graminis* f.sp. *tritici*, a basidiomycete) encodes a family of kinases, comprised of 14 members, with similar protein size. These show distant sequence similarity to the presumptive *Bgh* kinase family (E values 1e-04 to 1e-20) and may represent the basidiomycete equivalent of the ascomycete pathogen-associated kinases. It remains to be seen whether these proteins are active kinases, what their substrates are and whether and how they contribute to pathogenic processes.

### *Bgh*- and powdery mildew-specific proteins

We identified 1,072 *Bgh* proteins without significant NCBI BLASTP hits (E value ≥1e-06) except from hits to other *Bgh* proteins (Additional file 1: Table S1, sheet “Bgh BLAST hits only”). These include the 830 proteins without any considerable BLASTP hit mentioned above (Additional file 1: Table S1, sheet “No BLAST hit”) plus 224 EKA-like proteins and 18 proteins with solely *Bgh*-specific BLASTP hits. In total, the 1,072 proteins comprise 487 CSEPs, 231 EKA-like proteins and otherwise mainly unknown/hypothetical proteins. Only 77 of the 1,072 proteins contain an InterProScan domain, IPR016191 (ribonuclease/ribotoxin) being present 61-times, while all other domains occur only once or twice. This finding is consistent with the previous analysis of *Bgh* CSEPs, which uncovered unexpected affinities of a subset of this protein group to microbial ribonucleases [7]. TBLASTN analysis reveals that 303 of the proteins are also encoded by the *E. pisi* genome (E value <1e-10), while 278 are encoded by the *G. orontii* genome (E value <1e-10) as well. In total, 266 proteins, the majority thereof EKA proteins but also including twelve CSEPs, are common to all three powdery mildews and thus seem to represent powdery mildew-specific proteins (Additional file 1: Table S1, sheet “Powdery mildew-specific proteins”). On the other hand, 709 proteins appear to be *Blumeria*/*Bgh*-specific proteins since recognizable orthologs (E value <1e-5) are absent in both *E. pisi* and *G. orontii* (Additional file 1: Table S1, sheet “Bgh-specific proteins”). These 709 *Blumeria*/*Bgh*-specific proteins include 445 of the previously described 491 CSEPs [7].

### Proteins conserved in and specific to filamentous fungi

We recently found that the genomes of powdery mildew fungi lack a core set of 99 genes that are otherwise conserved in fungal species from yeasts to filamentous fungi [3]. These and possibly further missing genes could be the molecular cause for the obligate biotrophic lifestyle of these parasites. We speculated that the actual number of “missing genes” in powdery mildews might be considerably higher, since we applied very strict criteria for the automated BLAST searches, which would exclude all borderline cases that could for example arise from the presence of similar domains in otherwise unrelated proteins. To obtain a first insight whether this hypothesis was correct, we analyzed the powdery mildew genomes for the presence of 37 fungal ortholog MCLs that were previously found to be present exclusively in a set of 25 analyzed genomes of filamentous fungi, but not in the genomes of seven tested unicellular fungi (yeasts; [18]). Of the 37 MCLs analyzed, eight (22%) were not represented in the *Bgh* genome (Table 2). Thus, the analysis of this diagnostic set of proteins that are otherwise conserved in and specific to filamentous fungi confirms our hypothesis that additional genes have been lost in the powdery mildews.

**Table 2:** **Proteins conserved in and specific to filamentous fungi that are absent in the** ***Bgh*** **proteome** ^**a**^

MCL ID ^b^	Annotation (accession number) ^b^
MCL94	O-methylsterigmatocystin oxidoreductase (cytochrome P450) (O13345)
MCL1912	Neutral/alkaline non-lysosomal ceramidase (PF04734; Afu1g06470)
MCL2061	Homogentisate 1,2-dioxygenase (Q00667)
MCL2812	Vegetatible incompatibility protein HET-E-1 (Q00808)
MCL3026	Saccharopine dehydrogenase (Q8R127)
MCL3518	Similar to human LRP16 (Q9BQ69)
MCL3670	Ketosamine-3-kinase (Q8K274)
MCL4033	Citrate lyase beta chain (O53078)

^a^based on BLASTP searches against the *Bgh* proteome and TBLASTN searches against the *Bgh* genome; BLAST searches had to be either negative (E value >1e-10) or when positive were inspected manually for protein identity.

^b^according to [18].

### Proteins crucial for the formation of hyphal anastomoses

Hyphal anastomoses (somatic cell fusions during vegetative growth) are common in filamentous fungi. They are typically formed during colony expansion and contribute to the development of an extensive interconnected mycelium [19]. Somatic fusion of fungal cells often occurs early during fungal development and involves dedicated conidial anastomosis tubes (CATs; [20]). Formation of anastomoses can take place within a fungal colony and between fungal colonies. In the case of genetically diverse colonies, the latter can result in a heterokaryon, i.e. the presence of genetically different nuclei in a common cytoplasm [19]. Genetic analysis revealed a few factors that are required for anastomosis formation in the model ascomycete *N. crassa* [21]. These encode transcription factors, components of signal transduction and proteins involved in vesicle trafficking and membrane fusion [21].

To our knowledge, formation of hyphal anastomoses has never been reported for powdery mildews, suggesting that these ascomycetes are incapable of developing this type of cell-cell connections. To find out whether the apparent inability to generate somatic cell fusions is correlated with the absence of genes known to be required for anastomosis formation, we analyzed the presence/absence of these genes in the powdery mildew genomes. We found that of the 24 genes analyzed, two (*ada-3* and *so*/*ham-1*) were absent in the genomes of all three powdery mildew species (*Bgh*, *E. pisi* and *G. orontii*). By contrast, these two genes were present in the genomes of the closely related fungal species *B. fuckeliana* and *S. sclerotiorum*, which are both capable of forming hyphal anastomoses [22, 23]. In conclusion, these findings suggest that powdery mildews possibly lack the ability to form hyphal anastomoses owing to a lineage-specific loss of at least two genes coding for proteins that are essential for this process.

### Analysis of protein families with prominent roles in fungal development, signaling and pathogenesis

To find out whether additional fungal pathways that are known to play important roles in fungal development, signaling and pathogenesis are affected by genes not present in *Bgh*, we conducted manual inspection of a number of key pathways, comprising heterotrimeric G protein signaling, mitogen-activated protein kinase (MAPK) signaling, cyclic adenosine monophosphate (cAMP) signaling, small G protein signaling, calcium signaling and transcription factors (TFs).

### Heterotrimeric G protein signaling

G protein coupled receptors (GPCRs) are heptahelical cell surface receptors that, upon extracellular binding of a cognate ligand, initiate intracellular signal transduction pathways *via* heterotrimeric G proteins. In fungi, GPCRs sense diverse extracellular signals including: pheromones, carbohydrates, amino acids, nitrogen sources and photons [24, 25]. Together with the associated downstream heterotrimeric G protein signaling complex they engage in essential functions during growth, asexual and sexual development and virulence in the case of pathogenic fungi. In contrast to mammals, where GPCRs form a large and sequence-diversified family with several hundred members per species [26], most fungal genomes encode few GPCR proteins (often less than ten) and a limited set of heterotrimeric G protein components (typically three Gα subunits, one Gβ and one Gγ subunit; [24, 25]. Notably, species of the ascomycete subphylum Pezizomycotina encode a type of GPCR (PTH11) not found in other fungal groups. This type of GPCR is present as an expanded family of 61 members in the phytopathogen *M. grisea* [27]. Regulator of G protein signaling (RGS) proteins control the activity of Gα subunits [24], while phosducin proteins regulate Gβγ subunits, possibly by serving as chaperones [24]. Therefore these two types of proteins represent important rheostats of G protein signaling pathways.

**Figure 3:**
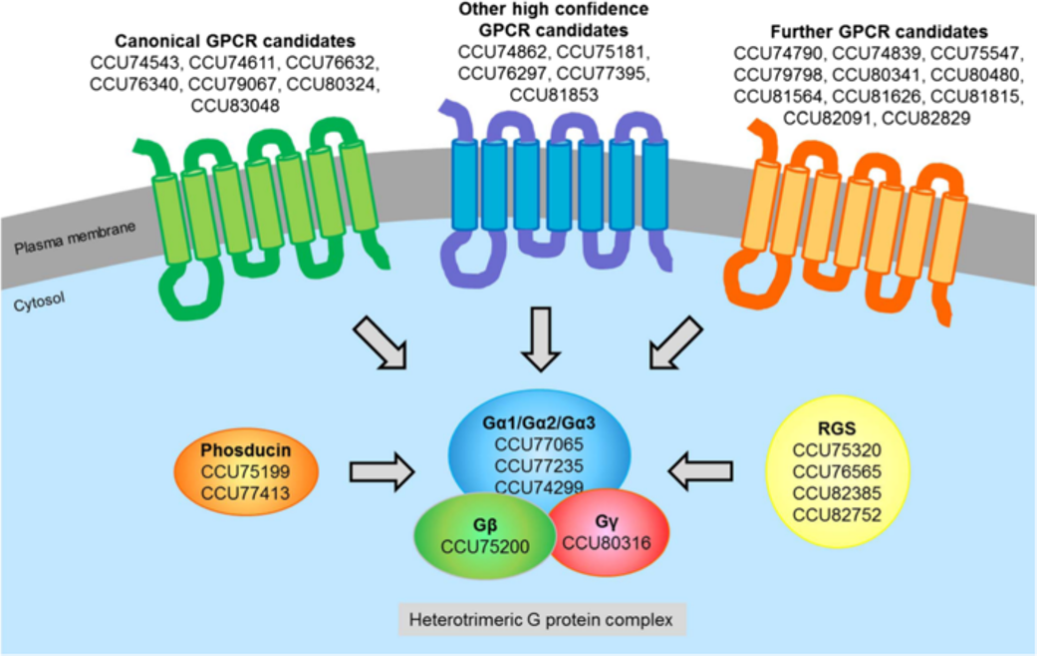
**Heterotrimeric G protein signaling components conserved in** ***Bgh*** **.** The scheme depicts GPCR candidates, heterotrimeric G protein subunits and regulatory components of GPCR signaling (phosducin, RGS). Note that the Ste3-like pheromone receptor is not shown since it is not represented in the current genome assembly of the *Bgh* DH14 isolate, but present in the transcriptome assemblies of *Bgh* isolates A6 and K1. One “Further GPCR candidate” and one RGS protein whose gene models are split on two contigs are also not included in the Figure. For further details see Additional file 7: Table S5 (sheet “GPCRs-heterotrimeric G proteins”).

We mined the *Bgh* genome for the presence of genes coding for heterotrimeric G protein subunits, GPCRs, RGS and phosducin proteins. We identified the archetypal set of heterotrimeric G protein components (three Gα subunits, one Gβ and one Gγ subunit), GPCRs (eight canonical GPCRs, including: putative pheromone, carbohydrate, nitrogen, cAMP and light receptors) and as well as five RGS and two phosducin proteins (Figure 3; Additional file 7: Table S5, sheet “GPCRs-heterotrimeric G proteins”). We identified one additional *Bgh* gene (besides the one encoding the regular Gβ protein) that encodes a protein harboring InterProScan domain IPR001632 (G protein beta WD40 repeat; CCU74556). This domain is characteristic of Gβ subunits; however, it still remains to be seen whether the respective protein, which is also encoded in the genomes of many other ascomycetes, has an authentic role in heterotrimeric G protein signaling. While three of the five predicted RGS proteins are well-conserved in other fungi (CCU76565, CCU75320, plus one corresponding to yeast Sst2, which is split on two genomic contigs and thus no GenBank accession number available), the other two (CCU82385, CCU82752) show limited sequence conservation and thus represent less common or less preserved types of RGS. The domain architecture of CCU75320 resembles human RGS-PX1, a presumably bifunctional protein in which the RGS domain is associated with sorting nexin features, thereby possibly linking G protein regulation to vesicular trafficking [28]. The genomes of many ascomycetes and higher plants encode a protein representing a presumed translational fusion of a GPCR with an RGS protein (*Aspergillus nidulans* GprK being the fungal prototype; [24, 29, 30]). Such a gene is absent in *Bgh*, but also missing in the genomes of *B. cinerea* and *S. sclerotiorum*, suggesting that this gene might have been lost in the lineage of the Leotiomycetes. Besides the eight canonical GPCRs, we found five additional high confidence GPCR candidate gene products based on the presence of characteristic InterProScan domains (including one homolog of *M. grisea* PTH11). Moreover, we identified twelve genes encoding proteins that, according to their predicted transmembrane topology and lack of homology to characterized proteins, may also represent GPCRs (Figure 3; Additional file 7: Table S5, sheet “GPCRs-heterotrimeric G proteins”). Either way, the number of GPCRs in *Bgh* seems to be considerably smaller than the extended GPCR complement found in the hemibiotrophic phytopathogen *M. grisea* [27].

### MAPK signaling

**Figure 4:**
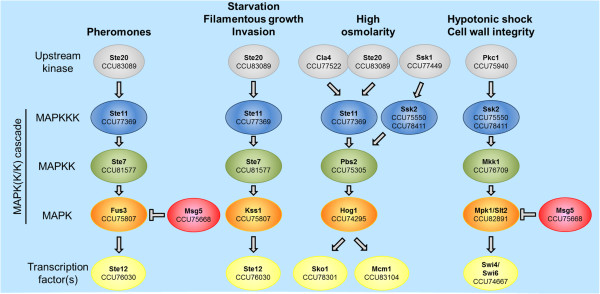
**Predicted MAPK signaling cascades in** ***Bgh*** **.** The scheme illustrates tentative MAPK signaling modules on the basis of known yeast MAPK cascades [37]. For further details, see Additional file 7: Table S5 (sheet “MAP kinases”).

Mitogen activated kinases (MAPKs) are highly conserved eukaryotic protein kinases. They typically function in modules of three-tiered signaling cascades, in which MAPKs are phosphorylated and activated by MAPK-kinases (MAPKKs), which in turn are phosphorylated and activated by MAPKK-kinases (MAPKKKs). MAPKKKs are connected to cell surface sensors *via* small monomeric GTPases and/or other upstream protein kinases. MAPK signaling is required for appressorium formation in several phytopathogenic and entomopathogenic fungi [31–35]. Analysis of the *Bgh* proteome revealed the presence of four canonical MAPKKKs (CCU75550, CCU77369, CCU78411 and CCU82598,), three prototypical MAPKKs (CCU75305, CCU76709 and CCU81577) and three archetypal MAPKs (CCU74295, CCU75807 and CCU82891; Additional file 7: Table S5, sheet “MAP kinases”). These proteins harbor characteristic kinase domains (for example PTHR24355, PTHR24360, PTHR24361 or IPR003527), and comprise presumptive orthologs of key MAP(K/K)Ks that are known to have important roles in fungal signaling and development (Figure 4; [36]). In addition to these classical MAP(K/K)Ks, the presence of distinctive protein domains suggests five additional MAPKKKs and three further MAPKs in *Bgh*. However, some of these proteins appear to be kinases acting upstream of MAPK modules (for example CCU77522 and CCU83089, corresponding to yeast Cla4 and Ste20, respectively) or kinases that have sequence similarity to MAPKKKs/MAPKs, but may not necessarily exert this function.

Based on the compilation of [37], we also studied the presence/absence of genes that encode proteins known to act upstream or downstream of MAP(K/K)K signaling. All signaling components typically present in filamentous fungi were found to be present. These include some known upstream kinases (corresponding to yeast Ste20, Cla4, Pkc1 and Ssk1) and downstream transcription factors (corresponding to yeast Mcm1, Sko1, Ste12 and Swi4/Swi6). Thus, the canonical MAPK signaling pathways as found in yeast appear to be complete in *Bgh* (Figure 4). The additionally identified MAPKKK and MAPK candidates leave, however, room for combinatorial modifications of the existing cascades or novel types of MAPK modules.

Two of the three canonical *Bgh* MAPKs identified by our genome-wide search (CCU75807 and CCU82891; corresponding to MAPK-I and MAPK-II, respectively) have been previously described and analyzed [38]. Another experimental study revealed a rapid transient increase in MAPK activity during early development of *Bgh* sporelings (appressorial germ tube formation and differentiation of appressoria) on cellulose membrane. In addition, exogenous application of activators of MAPK signaling (sphingosine and PAF-16) promoted fungal development on this artificial surface, while a MAPK inhibitor (PD 98095) had the opposite effect [39]. Thus, similar to other phytopathogens [31, 33, 40, 41], MAPK signaling in *Bgh* appears to play a role in the differentiation of key infection structures.

### cAMP signaling

**Figure 5:**
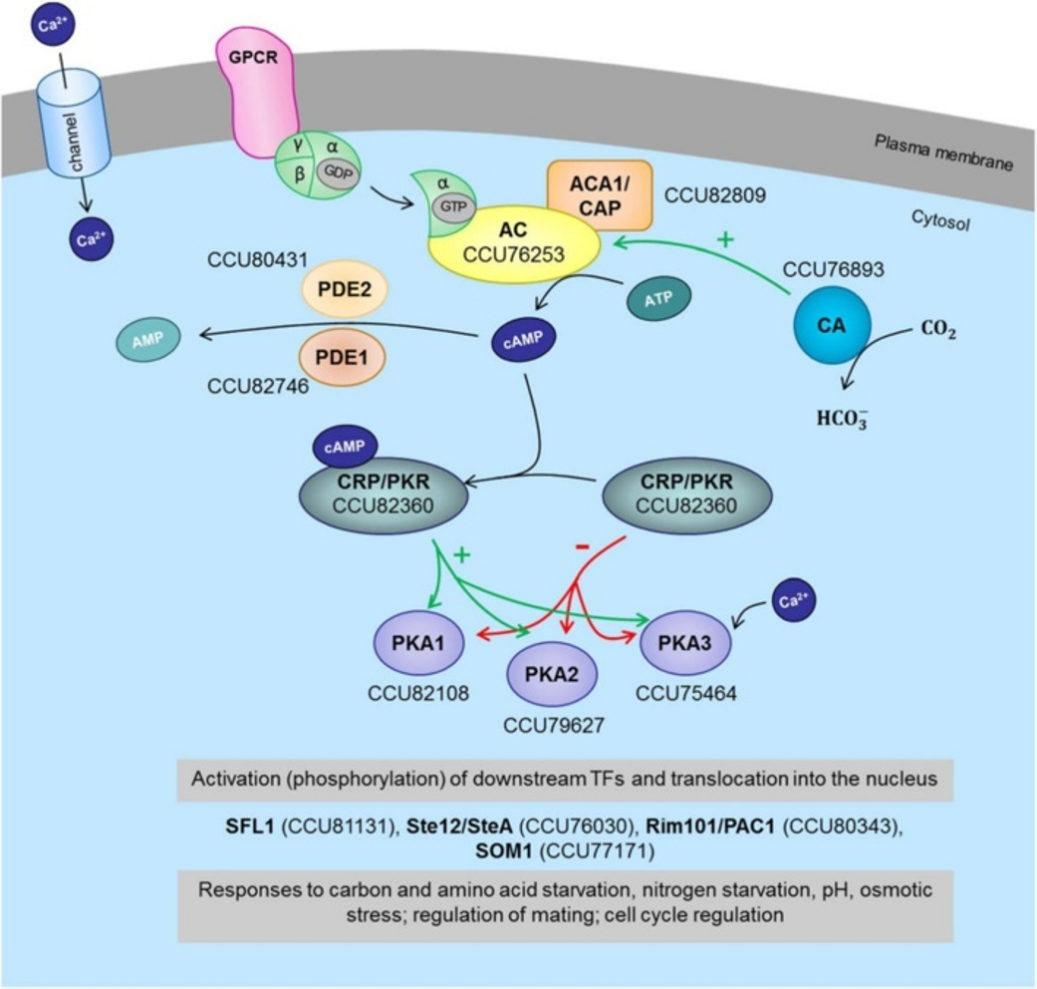
**cAMP signaling components conserved in** ***Bgh*** **.** The scheme was drawn after searching the *Bgh* genome for proteins with a described or predicted role in cAMP signaling in fungi [42]. AC, adenylate cyclase; ACA1/CAP, adenylate cylase-associated protein; AMP, adenosine monophosphate; ATP, adenosine triphosphate; CA, carbonic anhydrase; CRP/PKR, cAMP receptor protein/protein kinase A regulatory subunit; PDE1/PDE2, phosphodiesterase 1/2; PKA1/2/3, cAMP-dependent protein kinase A1/2/3 (catalytic subunit). For further details see Additional file 7: Table S5 (sheet “cAMP signaling”).

The cAMP pathway is a highly conserved central node of fungal development and virulence. It is responsive to nutrient and oxidative stress and regulates growth, cell cycle and pathogenesis in fungi [42–45]. The pathway is essentially comprised of adenylate cyclase, which forms cyclic AMP (cAMP) from ATP and is activated by stress-responsive upstream components, and protein kinase A (PKA), which acts as a heterotetramer composed of two catalytic subunits and two cAMP-binding regulatory subunits. PKA activates, in turn, transcriptional regulators that control expression of stress-responsive and cell cycle-associated genes (Figure 5). In our analysis we found that *Bgh* possesses all components involved in cAMP signaling, i.e. adenylate cyclase (CCU76253), an adenylate cyclase-associated protein (CCU82809), three types of catalytic PKA subunits (CCU79627, CCU82108 and CCU75464) and one regulatory PKA subunit (CCU82360), as well as two cyclic nucleotide phosphodiesterases (CCU82746 and CCU80431), which convert cAMP back to its non-cyclic form. Additionally, genes encoding several known downstream targets of PKA, such as Ste12/SteA (a transcription factor involved in fungal sexual reproduction; CCU76030), SFL1 (a flocculation suppression protein; CCU81131) and Rim101/PAC1 (a pH-responsive transcription factor; CCU80343) are also represented in the *Bgh* genome. Accordingly, the cAMP pathway seems to be complete in *Bgh* (Figure 5).

The findings from our *in silico* genomic analysis are consistent with previous experimental studies, which support a role for cAMP signaling in *Bgh* pathogenesis. For example, a rise in *Bgh* cAMP levels correlates with conidial differentiation on the host surface, while cAMP levels remain unaltered on a non-inductive glass surface [46]. Moreover, exogenous application of cAMP analogs and pharmacological stimulation and inhibition of PKA further support a role for cAMP signaling in conidial differentiation and appressorial development in *Bgh* [39, 47, 48].

### Small monomeric GTPase signaling

Members of the small monomeric GTPase superfamily play crucial roles in many cellular processes, including: signaling, endomembrane trafficking, cell cycle regulation and protein transport through the nuclear pore complex [49]. In *Bgh*, we found 33 small GTPases, belonging to the canonical Ras-, Ras-like-, Rab-, Ran-, and Rho-families, and 6 Arf-type GTPases (Additional file 7: Table S5, sheet “Small monomeric GTPases”). None of the families shows abnormalities concerning number of members or presence/absence of essential small GTPases. Furthermore, three mitochondrial small GTPases were found in the *Bgh* genome. Two of these belong to the Ras superfamily, whereas the other one corresponds to the Rho-type GTPase Miro1/2 (Gem1p in yeast; *Bgh* CCU80313), a calcium-dependent mitochondrial GTPase ubiquitously present in eukaryotes [50]. As described for Gem1p, *Bgh* Miro1/2 harbors two predicted GTPase domains and EF-hand like calcium binding domains. Numerous genes coding for corresponding GTPase activating proteins (GAP) and Guanine nucleotide exchange factors (GEF) are present in the *Bgh* genome. *Bgh* possesses 26 GAPs and 5 ArfGAPs, the latter being involved in vesicle fusion and fission, 16 GEFs, and 4 ArfGEFs (Additional file 7: Table S5, sheet “Small monomeric GTPases”). Taken together, all canonical small GTPase families are represented in the *Bgh* genome.

### Calcium signaling

**Figure 6:**
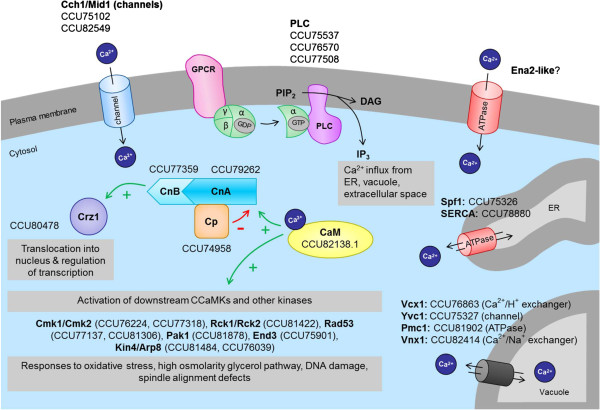
**Ca** ^**2+**^ **signaling components conserved in** ***Bgh*** **.** The scheme was drawn after searching the *Bgh* genome for proteins with a described or predicted role in Ca^2+^ signaling in fungi [37, 52, 53]. CaM, calmodulin; CnA/CnB, calcineurin A/B; Cp, calcipressin; Crz1, calcineurin-responsive zinc finger transcription factor; DAG, diacylglycerol; PLC, phospholipase C; IP_3_, inositol-1,4,5-trisphosphate; PIP_2_, phosphatidylinositol-4,5-bisphosphate; SERCA, SERCA-type Ca^2+^-transporting P-type ATPase. For further details see Additional file 7: Table S5 (sheet “Calcium signaling”).

The secondary messenger calcium (Ca^2+^) is involved in the regulation of a variety of cellular processes in eukaryotes. In filamentous fungi, Ca^2+^--mediated signal transduction is linked to responses to environmental stress as well as the regulation of fungal development including: spore germination, appressorium formation, polar growth, hyphal branching and sporulation (reviewed in [37, 51]). The perception of extracellular signals results in a transient increase in the cellular concentration of free Ca^2+^, often mediated *via* activation of phospholipase C (PLC) by GPCRs. PLC subsequently hydrolyses the membrane phospholipid PIP2 to form IP3 and diacylglycerol (DAG), two secondary messengers that initiate Ca^2+^ fluxes from the ER, vacuole and extracellular space. The resulting increase of cytoplasmic Ca^2+^ concentration is a prerequisite for activation of a number of diverse downstream signaling components for example kinases, including protein kinase C (PKC), Ca^2+^/calmodulin and (CaM)-dependent kinases (CCaMKs). The tight regulation of cytosolic Ca^2+^ concentration requires the activity of several cellular components (Figure 6; [51, 52]).

In resting eukaryotic cells, the intracellular Ca^2+^ concentration is maintained at a remarkably low level and increases upon extracellular cues due to the activity of Ca^2+^ pumps and transporters located in the plasma membrane (PM), ER membrane or the tonoplast [51]. In *Bgh* we found putative homologs for most proteins known or predicted to be involved in transmembrane Ca^2+^ transport, including: Ca^2+^ channels (Yvc1, Cch1/Mid1), ATPases (Spf1, SERCA-like, Pmr1, Pmc1) and Na^2+^/Ca^2+^ exchangers (Vnx1, Ecm27) (Figure 6, Additional file 7: Table S5, sheet “Calcium signaling”; [52, 53]). However, no obvious homolog could be identified for the plasma membrane-resident yeast Ca^2+^ P-type ATPase Ena2, which is conserved in *N. crassa* and *M. oryzae* and in the latter critical for appressorium formation [52]. On the other hand, an additional putative Ca^2+^ ATPase not present in yeast was identified (CCU78880). This protein is related to the endoplasmic/sarcoplasmic Ca^2+^P-type ATPases IIa of the SERCA type, which is conserved in *M. oryzae* and *N. crassa* and involved in virulence of the human pathogenic basidiomycete *Cryptococcus neoformans* [54]. Ca^2+^ ion fluxes from the environment or intracellular organelles precede the binding of Ca^2+^ to calmodulin (CaM/calcium-modulated protein), which transduces Ca^2+^ signatures by Ca^2+^-mediated interactions with various target proteins. CaM is present in all eukaryotes and usually highly conserved. Interestingly, we found a relatively low degree of CaM conservation between yeast and *Bgh* (60% amino acid identity, E value 2e-58). The best BLAST hit for *Bgh* CaM (CCU82138) is found in *Metarhizium robertsii*, an entomopathogenic, insect-infecting ascomycete (99% amino acid identity, E value 3e-94, EXV03624).

Subsequently, CaM interacts with and activates a variety of downstream acting proteins including calcineurin (Cn), a protein phosphatase that controls morphogenesis and stress responses in eukaryotes. Fungal pathogens have adopted the calcineurin pathway to survive and effectively propagate within the host (reviewed in [37, 51]). Calcineurin is formed by a heterodimer of a catalytic subunit (CnA) and a regulatory subunit (CnB) and is further regulated by calcipressin (Cp), which in yeast is represented by the Calcipressin-1 Rcn1p that is not conserved in *Bgh*. However, BLAST searches with mammalian Cp-2 and Cp-3 family members as query identified the *Bgh* protein CCU74958 as a possible candidate for calcipressin, albeit with low amino acid identity (ca. 38% to human calcipressin isoforms). In accordance with this finding, InterProScan predicts the calcipressin motif IPR006931 for the *Bgh* protein. In yeast, active calcineurin dephosphorylates the transcription factor Crz1, which subsequently enters the nucleus [55]. Nuclear Crz1 binds to calcineurin-dependent response elements (CDRE) and activates its own transcription as well as target genes linked to cell wall synthesis and Ca^2+^ homeostasis including: Pmr1, Pmc1, Cch1, Ena1 [56].

### Transcription factors

Transcription factors (TFs) are essential components in signal transduction, linking signal flow to transcriptional output. They are thus crucial mediators in fungal development and pathogenesis. Fungi contain dozens of TF families, including some seemingly fungus-specific types of TF families [57]. We inspected the meta-analysis data of TFs provided by the Fungal Transcription Factor Database (FTFD; http://ftfd.snu.ac.kr/index.php?a=view; [58]) and compared the results of the semi-automated classification provided in this database to respective data sets from the closely related species *B. cinerea* and *S. sclerotiorum*. According to this analysis, which is based on 5,473 open reading frames, the *Bgh* genome encodes 240 TFs that can be classified into 36 families, representing ca. 4.4% of the *Bgh* ORFs (Additional file 7: Table S5 (sheet “Transcription factors”). These numbers compare to 454 TFs (43 families; 2.8%) in *B. cinerea* and 648 TFs (44 families; ca. 4.5%) in *S. sclerotiorum*, respectively. The overall average for the ratio of transcription factors to all ORFs for fungi in the FTFD database is 4.6% (on the basis of 178 fungal species/strains), indicating that the relative number of TFs in *Bgh* is in the common range (Additional file 7: Table S5 (sheet “Transcription factors”). Although the FTFD database indicates that some TF families might be absent in *Bgh* (including: Forkhead TFs, GATA-type zinc finger TFs, Homoeobox TFs, MADS box TFs, negative transcriptional regulators and BED-type zinc finger TFs), manual inspection revealed that members of most of these families are in fact encoded by the *Bgh* genome and were possibly excluded or ignored by the automated FTFD classification pipeline. Exceptions are the negative transcriptional regulators - a class of TFs that seems indeed to be absent in *Bgh*. The prototype of this family is NmrA, a negative transcriptional regulator involved in metabolite (for example nitrogen) repression in various fungi, including *N. crassa* and *A. nidulans* [59]. Since *Bgh,* as a highly host-adapted obligate biotrophic pathogen, does not rely on alternative nitrogen sources, such regulatory circuits might be dispensable.

## Conclusions

Our study reports basic characteristics of the *Bgh* genome (Figure 1, Table 1, Additional file 1: Table S1). Apart from the largest protein families, which revealed *Bgh*-specific attributes (CSEPs and EKAs), most features are comparable to other fungal genomes. However, consistent with our hypothesis that the list of missing genes in *Bgh* might be incomplete, our analysis uncovered a number of protein coding genes lacking in *Bgh* compared to other (filamentous) fungi. These include: proteins, specific to filamentous fungi (Table 2), which are required for anastomosis formation, negative transcriptional regulators and individual components in common signaling pathways. By contrast, all main components of major signal transduction pathways (heterotrimeric G protein signaling, MAPK signaling, cAMP signaling, Ca^2+^ signaling, small GTPases) are present, suggesting that these pathways are functional in *Bgh* (Figures 3, 4, 5 and 6). We identified a set of powdery mildew- and *Bgh*-specific genes and found that a family of pathogenesis-related kinases with unknown kinase specificity is unusually expanded in *Bgh* (Figure 2, Additional file 6: Table S4). The functional relevance of these kinases for the *Bgh* life cycle remains to be discovered.

## Methods

### Protein sequences

The 6,470 protein sequences deduced from the annotated *Bgh* isolate DH14 genome (v3.0) (http://www.blugen.org/) form the basis of this study. This core protein set was supplemented with 25 additional proteins that were annotated in the course of this analysis. GenBank accession numbers of all proteins can be found in Additional file 1: Table S1, except for the Ste3-like GPCR, which is missing in the genome assembly of isolate DH14. The latter protein sequence was deduced from the transcriptome of *Bgh* isolates A6 and K1 [5]. The EBI/GenBank accession numbers for 24 out of the 25 newly annotated sequences are CEA17182-CEA17205 (note that for the Ste3-like GPCR there is no accession number since the gene is missing in the genome assembly of isolate DH14).

### BLAST2GO analysis and BLAST searches

BLAST2GO (http://www.blast2go.com/b2ghome/about-blast2go) analysis [60, 61] was conducted in July 2013 using standard parameters (BLAST threshold set to 1e-06). *Bgh* BLAST searches were performed against the genomic assembly of *Bgh* isolate DH14 [3] and the transcriptome assemblies of *Bgh* isolates A6 and K1 [62]. BLAST searches against the genomes of *E. pisi* and *G. orontii* were performed on the basis of genomic draft assemblies (http://www.mpipz.mpg.de/23693/Powdery_Mildews) [3].

### Signal peptides and transmembrane domains

Prediction of signal peptides for secretion was performed with SignalP4.1 (http://www.cbs.dtu.dk/services/SignalP/; [63]), the presence of transmembrane domains was analyzed with TMHMM2.0 (http://www.cbs.dtu.dk/services/TMHMM/*;* [64]) and TOPCONS (http://topcons.cbr.su.se/; [65]).

### Markov clustering of *Bgh*proteins

A BLASTP [66] search was performed with each *Bgh* protein against the database containing all *Bgh* proteins, retaining all hits with an E value ≤ 1e-10. The results were converted into an abc file as input for the Markov clustering algorithm mcl (http://www.micans.org/mcl) [67] so that sequence similarities could be used as the basis for clustering. The inflation parameter 2 resulted in 4377 protein families. All data conversions and final data summaries were implemented in Perl scripts.

### Subcellular localization

Subcellular localization of proteins was inferred by ProtComp (Version 9, http://linux1.softberry.com). The resulting files were parsed with a Perl script. In cases where the neural net prediction was the same as reported with the highest integral score, the predicted location was assigned to the protein.

### Analysis of proteins conserved in, and specific to, filamentous fungi

Based on the MCL designations listed in the study by [18] we selected query protein sequences from the NCBI database to interrogate (local BLASTP and TBLASTN searches) the presence of genes encoding these proteins in the *Bgh* genome. In cases where BLAST results were inconclusive, manual inspection for the presence of informative domains *via* InterProScan was performed.

### Fungal signaling pathways

We analyzed prominent fungal signaling pathways in *Bgh,* including: heterotrimeric G proteins/GPCR, MAPKs, cAMP signaling, small monomeric GTPases and Ca^2+^ signaling, by performing sequence similarity searches. Query sequences were selected from other ascomycetes (for example *S. cerevisiae*, *S. pombe*, *N. crassa*, *C. neoformans*, and *M. oryzae*) on the basis of literature data [24, 37, 42, 52, 53, 68]. Putative *Bgh* orthologs of the query sequences were typically verified by reciprocal BLAST analysis and in many instances further corroborated by the presence of informative protein domains identified *via* InterProScan analysis. Additional GPCR candidates were identified by manual inspection of proteins with six to eight predicted transmembrane domains based on TMHMM2.0 and TOPCONS analysis (see Additional file 7: Table S5, sheet “GPCRs-heterotrimeric G proteins”).

## Availability of supporting data

The data sets supporting the results of this article are included within the article and its additional files.

## Electronic supplementary material

Additional file 1: Table S1: Global Analysis of the *Bgh* proteome. Sheet “Summary”: This sheet serves as a master sheet and contains all data regarding BLAST2GO analysis, GenBank accession numbers, protein description, SignalP4.1 analysis, TMHMM2.0 analysis, InterProScan results, BLAST results against the *G. orontii* and *E. pisi* genome and ProtComp analysis. Sheet “SignalP4.1 details”: Detailed output from the SignalP4.1 analysis. Sheet “No BLAST hit”: The 830 entries of the “Summary” sheet that have no BLAST hit. Sheet “Bgh BLAST hits only”: The 830 entries of the sheet “no BLAST hit” plus 224 EKA-like proteins plus 18 proteins with solely *Bgh*-specific BLAST hits (total of 1072 entries). Sheet “Fungus-specific kinase”: The 70 entries of the “Summary” sheet that correspond to the fungus-specific kinase. Sheet “CSEPs”: The 491 entries of the “Summary” sheet that correspond to the previously described CSEPs. Sheet “Additional effector candidates”: The 42 entries of the “Bgh BLAST hits only” sheet that have no BLAST hit and a prediction for a signal peptide by SignalP4.1. Sheet “Powdery mildew-specific proteins”: The 265 entries of the “Bgh BLAST hits only” sheet with TBLASTN hits E <1e-10 against both the *G. orontii* and the *E. pisi* genome. Sheet “Bgh-specific proteins”: The 709 entries of the “Bgh BLAST hits only” sheet with no TBLASTN hits E <1e-5 against both the *G. orontii* and the *E. pisi* genome. (XLSX 4 MB)

Additional file 2: Figure S1: BLAST hit distribution. Histograms showing the frequency distribution of BLAST hits with regard to species. The diagram is based on all BLAST hits (A) or only the top BLAST hits (B) obtained in BLAST2GO analysis (i.e., using the 6,495 *Bgh* proteins as a query). (PDF 17 KB)

Additional file 3: Table S2: *Bgh* protein family size distribution. (PDF 20 KB)

Additional file 4: Table S3: The 25 most abundant InterProScan domains found in the *Bgh* proteome. (PDF 22 KB)

Additional file 5: Figure S2: Prediction of subcellular localization by ProtComp. The pie charts illustrate the prediction profiles of subcellular protein localization obtained by “neural network analysis” (A) and the “integral final score” (B). Figures indicate the number of proteins falling into a given category. (PDF 22 KB)

Additional file 6: Table S4: Presence of a fungus-specific kinase family in selected fungal species. (PDF 37 KB)

Additional file 7: Table S5: *Bgh* protein families with prominent roles in fungal development, signaling and pathogenesis. Sheet “GPCRs-heterotrimeric G proteins”. Sheet “MAP kinases”. Sheet “cAMP signaling”. Sheet “Small monomeric GTPases”. Sheet “Calcium signaling”. Sheet “Transcription factors”. (XLSX 59 KB)

## Abbreviations

*Bgh*: *Blumeria graminis* f.sp. *hordei*
*Bgt*: *Blumeria graminis* f.sp. *tritici*
CSEP: Candidate Secreted Effector Protein
EKA: Effector with similarity to AVRK1 and AVRA10
GPCR: G protein coupled receptor
MAPK: Mitogen-activated protein kinase
TF: Transcription factor.
