# Supplementary figures and images for: In silico analysis of the core signaling proteome from the barley powdery mildew pathogen (Blumeria graminis f.sp. hordei)
Source: BMC Genomics. 2014 Oct 2;15(1):843. doi: 10.1186/1471-2164-15-843 (PMC4195978; doi:10.1186/1471-2164-15-843)

# Additional Figure 1

**A**

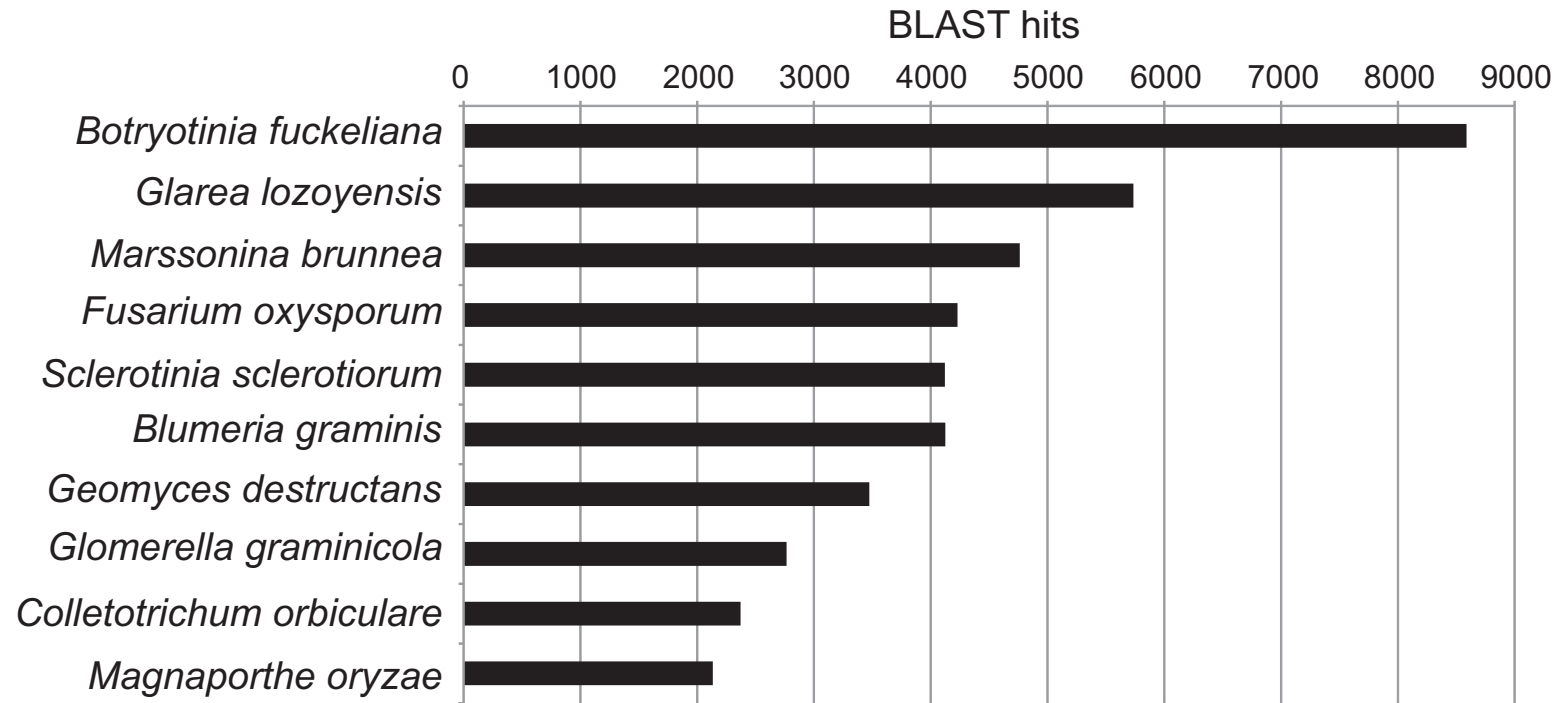

**B**

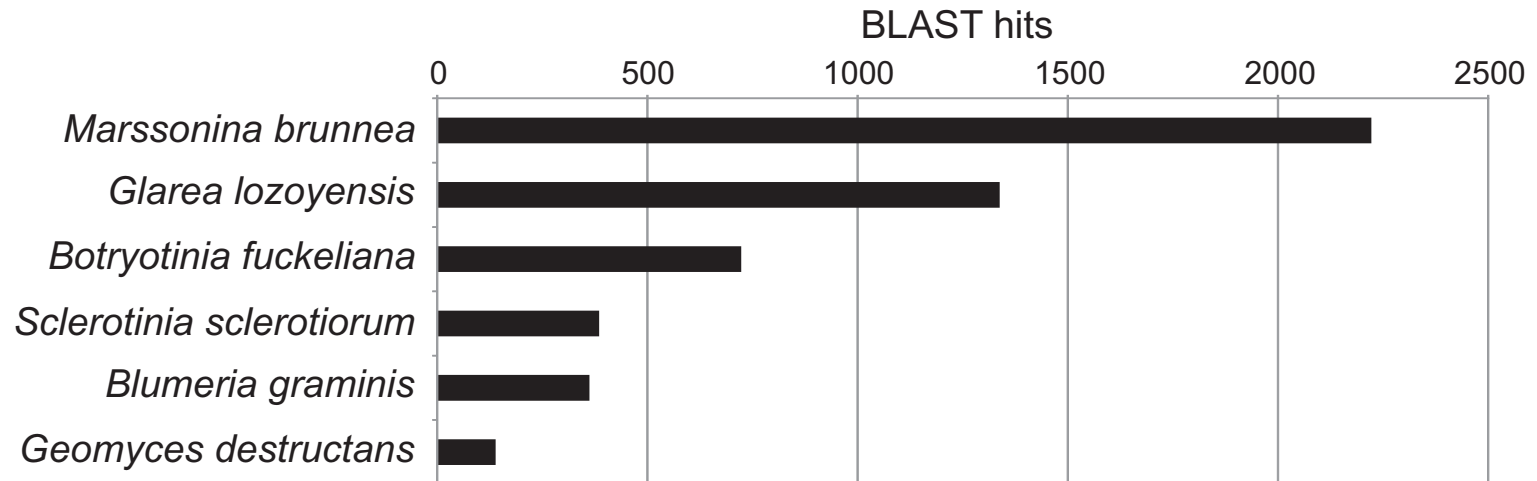

Supplement: Supplementary file 2 — Additional file 2: Figure S1: BLAST hit distribution. Histograms showing the frequency distribution of BLAST hits with regard to species. The diagram is based on all BLAST hits (A) or only the top BLAST hits (B) obtained in BLAST2GO analysis (i.e., using the 6,495 Bgh proteins as a query). (PDF 17 KB) [file 12864_2014_6527_MOESM2_ESM.pdf]

# Additional Figure 2

**A**

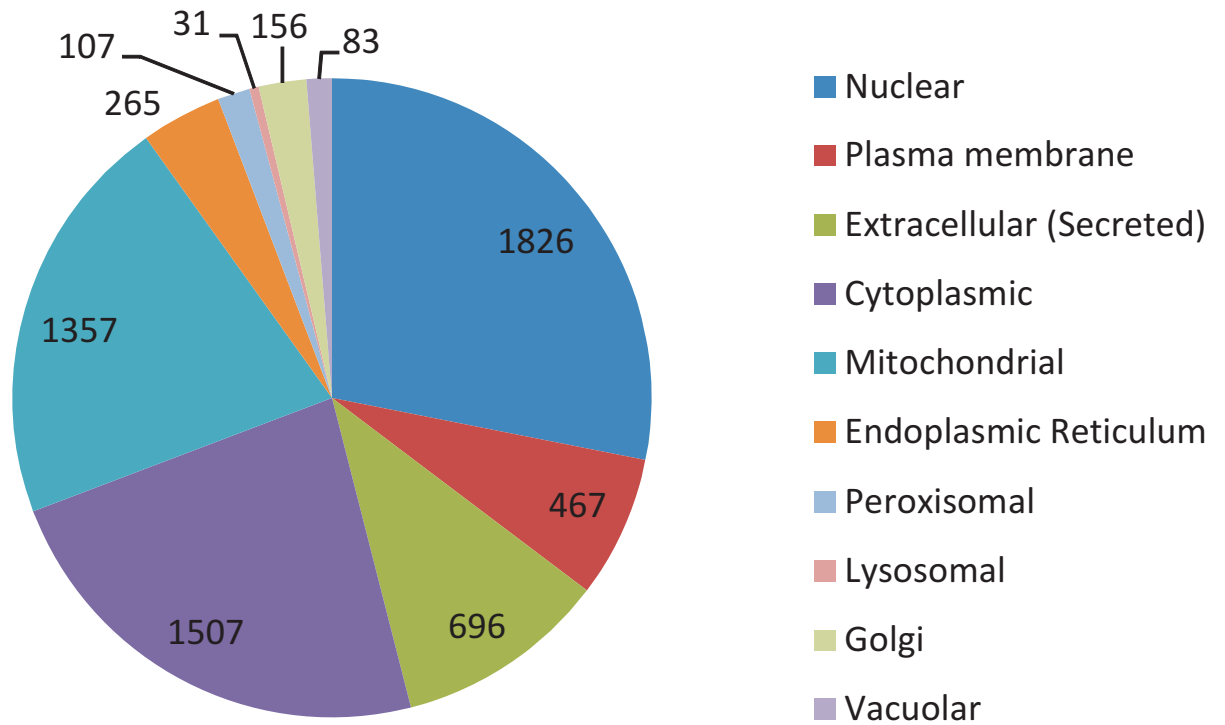

**B**

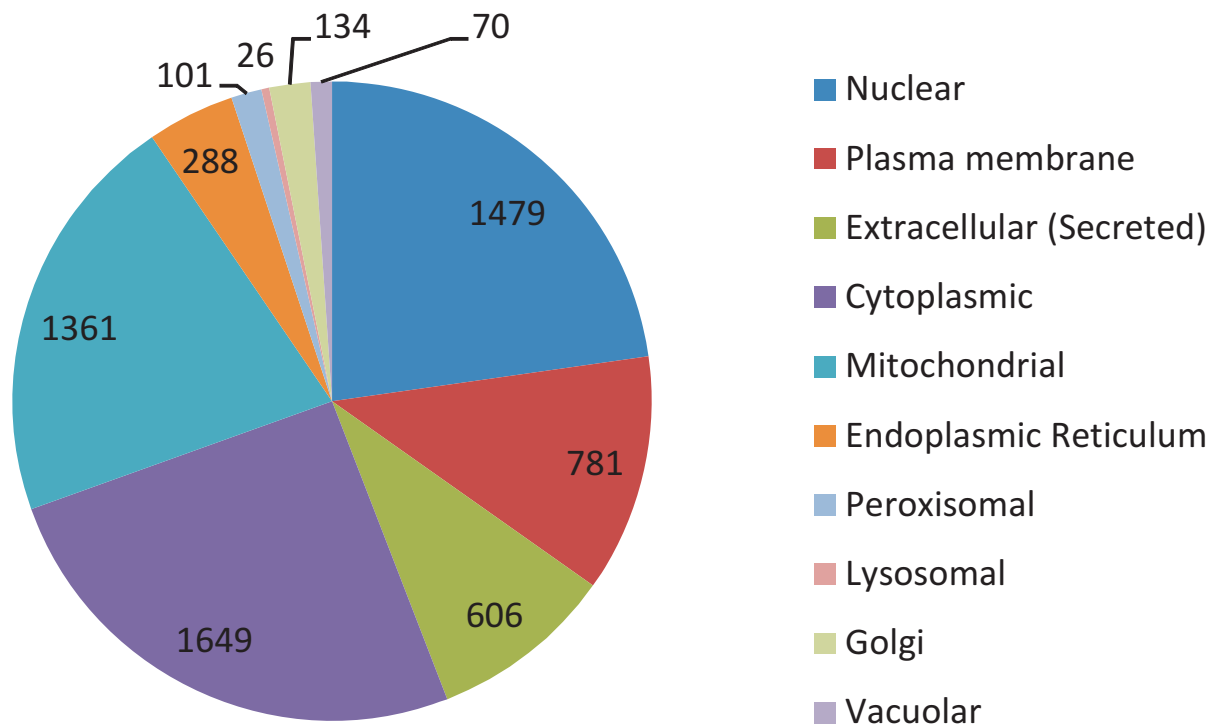

Supplement: Supplementary file 5 — Additional file 5: Figure S2: Prediction of subcellular localization by ProtComp. The pie charts illustrate the prediction profiles of subcellular protein localization obtained by “neural network analysis” (A) and the “integral final score” (B). Figures indicate the number of proteins falling into a given category. (PDF 22 KB) [file 12864_2014_6527_MOESM5_ESM.pdf]
