# Supplementary material for: In silico analysis of the core signaling proteome from the barley powdery mildew pathogen (Blumeria graminis f.sp. hordei)
Source: BMC Genomics. 2014 Oct 2;15(1):843. doi: 10.1186/1471-2164-15-843 (PMC4195978; doi:10.1186/1471-2164-15-843)
Supplement: Supplementary file 3 — Additional file 3: Table S2: Bgh protein family size distribution. (PDF 20 KB) [file 12864_2014_6527_MOESM3_ESM.pdf]

Additional file 3: Table S2. *Bgh* protein family size distribution.

Family size (number of proteins)	Frequency (number of families)
1	3,758
2	314
3	141
4	61
5	28
6	19
7	14
8	12
9	3
10	6
11-25	14
26-50	2
51-100	3
>100	2
