# Supplementary material for: In silico analysis of the core signaling proteome from the barley powdery mildew pathogen (Blumeria graminis f.sp. hordei)
Source: BMC Genomics. 2014 Oct 2;15(1):843. doi: 10.1186/1471-2164-15-843 (PMC4195978; doi:10.1186/1471-2164-15-843)
Supplement: Supplementary file 4 — Additional file 4: Table S3: The 25 most abundant InterProScan domains found in the Bgh proteome. (PDF 22 KB) [file 12864_2014_6527_MOESM4_ESM.pdf]

Additional file 4: Table S3. The 25 most abundant InterProScan domains found in the *Bgh* proteome.

Domain designation	Occurrence (-times) [a]	Description
IPR027417	305	P-loop containing nucleoside triphosphate hydrolase
IPR011009	149	Protein kinase-like domain
IPR015943	125	WD40/YVTN repeat-like-containing domain
IPR016024	102	Armadillo-type fold
IPR000719	100	Protein kinase, catalytic domain
IPR001680	93	WD40 repeat
IPR017986	88	WD40-repeat-containing domain
IPR016040	85	NAD(P)-binding domain
IPR002290	83	Serine/threonine- / dual-specificity protein kinase, catalytic domain
IPR020635	76	Tyrosine-protein kinase, catalytic domain
IPR008271	75	Serine/threonine-protein kinase, active site
IPR011989	75	Armadillo-like helical
IPR013083	72	Zinc finger, RING/FYVE/PHD-type
IPR003593	69	AAA+ ATPase domain
IPR001650	67	Helicase, C-terminal
IPR012677	67	Nucleotide-binding, alpha-beta plait
IPR014001	65	Helicase, superfamily 1/2, ATP-binding domain
IPR011990	60	Tetratricopeptide-like helical

IPR016196	60	Major facilitator superfamily domain, general substrate transporter
IPR019775	59	WD40 repeat, conserved site
IPR017441	54	Protein kinase, ATP binding site
IPR000504	47	RNA recognition motif domain
IPR012340	46	Nucleic acid-binding, OB-fold
IPR020846	46	Major facilitator superfamily domain
IPR011991	45	Winged helix-turn-helix transcription repressor DNA-binding

[a] Occurrence was scored independently of the hierarchical relationships of some domains to each other.
