# Supplementary material for: In silico analysis of the core signaling proteome from the barley powdery mildew pathogen (Blumeria graminis f.sp. hordei)
Source: BMC Genomics. 2014 Oct 2;15(1):843. doi: 10.1186/1471-2164-15-843 (PMC4195978; doi:10.1186/1471-2164-15-843)
Supplement: Supplementary file 6 — Additional file 6: Table S4: Presence of a fungus-specific kinase family in selected fungal species. (PDF 37 KB) [file 12864_2014_6527_MOESM6_ESM.pdf]

Additional file 6: Table S4. Presence of a fungus-specific kinase family in selected fungal species [a]

**Species**	**Ascomycete class**	**Fungal lifestyle**	**Number of hits (E value <1e-20) (stringent)**	**Number of hits (E value <1e-05) (relaxed)**
Blumeria graminis f.sp. *hordei* DH14 (taxid: 546991)	Leotiomycetes	Phytopathogen (powdery mildew)	62	70
Blumeria graminis f.sp. *tritici* (taxid 62690)	Leotiomycetes	Phytopathogen (powdery mildew)	19	25
Coccidioides immitis RS (taxid:246410)	Eurotiomycetes	Facultative human pathogen (coccidioidomycosis)	18	26
Claviceps purpurea 20.1 (taxid:1111077)	Sordariomycetes	Phytopathogen (ergot)	13	28
Ajellomyces dermatitidis ATCC 18188 (taxid: 653446)	Eurotiomycetes	Facultative human pathogen (blastomycosis)	11	17
Paracoccidioides brasiliensis Pb18 (taxid:502780)	Eurotiomycetes	Facultative human pathogen (paracoccidioidomycosis)	11	16

Macrophomina phaseolina MS6 (taxid:1126212)	Dothideomycetes	Phytopathogen (charcoal rot)	8	9
Metarhizium acridum CQMa 102 (taxid:655827)	Sordariomycetes	Entomopathogen (grasshopper)	6	10
Ajellomyces capsulatus H143 (taxid:544712)	Eurotiomycetes	Human pathogen (histoplasmosis)	5	10
Metarhizium anisopliae ARSEF 23 (taxid:655844)	Sordariomycetes	Entomopathogen	5	7
Sclerotinia sclerotiorum 1980 (taxid:665079)	Leotiomycetes	Phytopathogen	4	6
Beauveria bassiana ARSEF2860 (taxid:655819)	Sordariomycetes	Entomopathogen	4	5
Glarea lozoyensis ATCC 20868 (taxid:1116229)	Leotiomycetes		1	1
Neurospora crassa OR74A (taxid:367110)	Sordariomycetes	Saprophyte	1	1
Fusarium oxysporum f. sp. *cubense* (taxid:61366)	Sordariomycetes	Phytopathogen	0	2
Aspergillus niger ATCC 1015	Eurotiomycetes	Saprophyte	0	2

(taxid:380704)				
Magnaporthe oryzae 70-15 (taxid: 242507)	Sordariomycetes	Phytopathogen (rice blast)	0	0
Erysiphe pisi	Leotiomycetes	Phytopathogen (powdery mildew)	1	0
Golovinomyces orontii	Leotiomycetes	Phytopathogen (powdery mildew)	0	1
Saccharomyces cerevisiae (taxid: 4932)	Saccharomycetes	Saprophyte	0	0
Colletotrichum higginsianum (taxid:80884)	Sordariomycetes	Phytopathogen (anthracnose)	0	0
Botryotinia fuckeliana T4 (taxid:999810)	Leotiomycetes	Phytopathogen (gray mold)	0	0
Penicillium chrysogenum NCPC10086 (taxid:1297591)	Eurotiomycetes	Saprophyte	0	0
Marssonina brunnea f. sp. '*multigermtubi*' S1 (taxid:1072389)	Leotiomycetes	Phytopathogen (Marssonina leaf spot)	0	0
Stagonospora nodorum (taxid:13684)	Dothideomycetes	Phytopathogen (blotch)	0	0

[a] Based on BLASTP results using CCU82254 as a query sequence.
